# Preliminary evidence of mitochondrial dysfunction associated with post-infective fatigue after acute infection with Epstein Barr Virus

**DOI:** 10.1186/1471-2334-6-15

**Published:** 2006-01-31

**Authors:** Suzanne D Vernon, Toni Whistler, Barbara Cameron, Ian B Hickie, William C Reeves, Andrew Lloyd

**Affiliations:** 1 Centers for Disease Control and Prevention, 1600 Clifton Road, Atlanta, Georgia 30333, USA; 2 University of New South Wales, Sydney 2052, Australia; 3 Brain and Mind Research Institute, University of Sydney, New South Wales, Sydney 2006, Australia

## Abstract

**Background:**

Acute infectious diseases are typically accompanied by non-specific symptoms including fever, malaise, irritability and somnolence that usually resolve on recovery. However, in some individuals these symptoms persist in what is commonly termed post-infective fatigue. The objective of this pilot study was to determine the gene expression correlates of post-infective fatigue following acute Epstein Barr virus (EBV) infection.

**Methods:**

We followed 5 people with acute mononucleosis who developed post-infective fatigue of more than 6 months duration and 5 HLA-matched control subjects who recovered within 3 months. Subjects had peripheral blood mononuclear cell (PBMC) samples collected at varying time points including at diagnosis, then every 2 weeks for 3 months, then every 3 months for a year. Total RNA was extracted from the PBMC samples and hybridized to microarrays spotted with 3,800 oligonucleotides.

**Results:**

Those who developed post-infective fatigue had gene expression profiles indicative of an altered host response during acute mononucleosis compared to those who recovered uneventfully. Several genes including ISG20 (interferon stimulated gene), DNAJB2 (DnaJ [Hsp40] homolog and CD99), CDK8 (cyclin-dependent kinase 8), E2F2 (E2F transcription factor 2), CDK8 (cyclin-dependent kinase 8), and ACTN2 (actinin, alpha 2), known to be regulated during EBV infection, were differentially expressed in post-infective fatigue cases. Several of the differentially expressed genes affect mitochondrial functions including fatty acid metabolism and the cell cycle.

**Conclusion:**

These preliminary data provide insights into alterations in gene transcripts associated with the varied clinical outcomes from acute infectious mononucleosis.

## Background

Acute viral diseases such as infectious mononucleosis typically present clinically with a cluster of non-specific symptoms including; fever, an increased need to sleep, hyperalgesia, anorexia, loss of interest in usual activities, social interaction, body care, depressed mood, and impaired concentration [1-3]. This acute sickness behavior response comprises a highly organized and evolved disease-fighting strategy mediated by the action of pro-inflammatory cytokines [4-8]. In general, acute sickness behavior resolves in parallel with clearance or control of the infecting agent. However, some individuals exhibit prolonged illness with fatigue, mood changes and cognitive impairment. Such prolonged illness following infectious mononucleosis has been recognized for at least half a century [9]. Recent studies of infectious mononucleosis due to EBV infection demonstrated that fatigue, sore throat and malaise persisted for up to two months in approximately 40% of patients and for six or more months in approximately 10% [10,11]. The risk factors for and pathophysiology of this post-infective fatigue syndrome following EBV infectious mononucleosis have not been identified. It remains unclear whether post-infective fatigue reflects chronic effects of persistent EBV (e.g., viral-mediated) or whether acute EBV infection functions as a stressor that triggers an altered host response to the virus leading to ongoing symptoms.

The objective of this pilot study was to assess patients' gene transcription patterns in the peripheral blood following acute infectious mononucleosis due to EBV and to determine whether those who recovered uneventfully had different gene expression profiles than those who developed post-infective fatigue. To do this, a small cohort of HLA-matched individuals was followed over one year after infectious mononucleosis. We found that individuals who suffered from post-infective fatigue had a distinct gene expression profile during acute illness compared to those whose illness resolved. Evaluation of the gene expression profile over the course of the year implicated an altered host response to EBV and mitochondrial dysfunction in those who developed post-infective fatigue. These data provide insight into alterations in gene transcripts associated with the varied clinical outcomes from EBV-associated mononucleosis.

## Methods

This study adhered to human experimentation guidelines of the U.S. Department of Health and Human Services. All participants were volunteers who gave written informed consent.

Subjects were participants in a prospective cohort study based in the region surrounding the township of Dubbo in rural New South Wales, Australia. Subjects were enrolled following presentation to their general practitioner with symptoms suggestive of infectious mononucleosis and laboratory documentation of IgM antibodies against EBV viral capsid antigen (VCA). These provisional serological diagnoses were confirmed by repeated testing of longitudinally collected samples [12]. Most subjects were assessed at baseline, 2–3 weeks, 4–6 weeks, and 3 months. In those subjects with persistent symptoms, evaluation by both a physician and psychiatrist was undertaken at 6 months to exclude unrelated causes of ongoing illness. A late follow-up sample (12 months or longer after baseline) was collected from all subjects. At each visit, self-report and interview assessments of physical and psychological health were recorded and a blood sample was collected. A diagnosis of chronic fatigue syndrome (CFS) was made according to the international diagnostic criteria [13] in post-infective fatigue cases included in this study.

Those with post-infective fatigue were labeled as cases and those who recovered as controls. To control for the effect of genetic influences on immune responses, cases were matched to controls by HLA -A and -B genotype (with at least two, and up to four, matched HLA alleles). There were 5 cases with post-infective fatigue and 5 controls that recovered promptly. Subjects were also matched by sex and age. There were 3 females and 2 males in each group ranging in age from 16 to 49 years. Due to the small number of subjects, samples were grouped according to two categorized time periods: early (baseline to 3 months) and late (>6 months following disease onset). This resulted in 9 early and 8 late samples available from cases and 6 early and 6 late samples available from controls.

### Blood samples

Peripheral blood mononuclear cells (PBMC) were separated from blood by density gradient centrifugation (Lymphoprep, AXIS-SHIELD, Norway), cryopreserved in RPMI (Trace, Australia) with 10% DMSO (Sigma, Australia) and 50% autologous plasma, and aliquots stored in the vapor phase of liquid nitrogen. Total RNA was subsequently extracted using TRIzol (Invitrogen, Carlsbad, CA) and RNA integrity and quantity were determined by denaturing gel electrophoresis.

### Preparation and hybridization of labeled cDNA

Biotinylated cDNA synthesis from 1 μg of total RNA was performed as previously described [14]. The cDNA probe was hybridized to the Atlas™ Human 3.8I oligonucleotide glass microarrays (CLONTECH Laboratories, Inc., Palo Alto, CA) using the Ventana Discovery™ system and their ChipMap™ kit (Ventana Medical Systems, Tucson, AZ). Hybridization was for 8 hours at 42°C, followed by three 10 minute stringency washes, the final wash in 0.1× SSC at 42°C. Anti-biotin antibodies conjugated to RLS™ particles (Invitrogen, Carlsbad, CA) were used for signal detection as previously described [14]. The slides were archived and images captured using the GSD-501™ scanner (Invitrogen, Carlsbad, CA).

### Data analysis

The scanned TIFF images were processed using ArrayVision™ (Imaging Research Inc., Ontario, Canada) as previously described [15]. A median background value was calculated around each of the 3757 features and subtracted from the mean feature signal to give the net signal for the respective gene. Flagged features and features with a signal to noise ratio of <2.5 were excluded. There were 636 genes remaining with a valid signal in 80% or more of the arrays, that were then log2 transformed and the Z-score normalized [16].

The Class Comparison analyses were performed using BRB ArrayTools [17] developed by Dr. Richard Simon and Amy Peng Lam to determine if gene expression profiles were different between groups by applying a random variance t-test to the data. Genes were considered statistically significant with a median false discovery rate of 10%. The EASE software package [18] was then used to evaluate the biologic significance of the ontology of genes identified as differentially expressed by class comparison and to perform a statistical analysis of gene categories in the gene list to find those most over-represented in the arrays [19,20]. To demonstrate the distinct gene clusters, we performed a two-way hierarchical cluster analysis on the genes identified by this analytical approach, using the algorithm described by Eisen et al. [21]. The Reactome curated database [22] was used to map differentially expressed genes to biological processes in humans [23].

## Results

**Figure 1:**
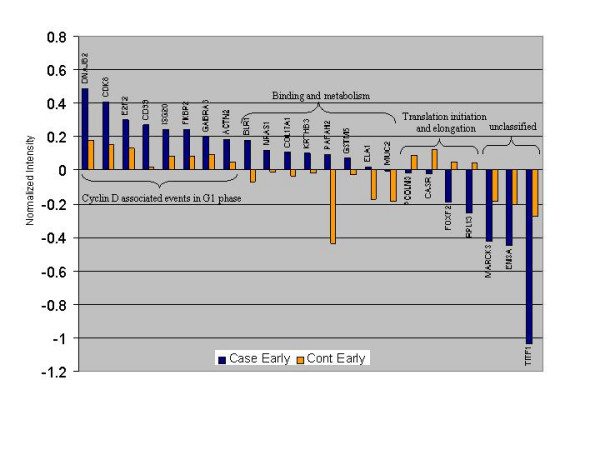
There were 23 of 636 genes that were expressed at different levels between cases (blue bars) and controls (orange bars) at the early time point. The majority of these (15/23) were expressed at higher levels in the cases compared to controls and most were involved in cell cycle and metabolism. The four genes known to be involved in translation, were expressed at much lower levels in the cases compared to controls.

All 10 subjects were followed for at least 12 months following acute EBV infection. To determine if there were differences between subjects who developed post-infective fatigue and those who recovered uneventfully, gene expression of early samples (baseline to 3 months) from cases and controls were compared. Twenty-three genes were differentially expressed during the early time point between cases and controls (Figure 1). Of these, 8 genes (35%) were more highly expressed on average in cases (blue bars) than in controls (orange bars). These over-expressed genes mapped to cyclin D associated events in G1 phase of the cell cycle. Four genes were more highly expressed in controls than in cases and all mapped to pathways involved in translation initiation and elongation. Eight genes were expressed only in cases and these were classified into binding and metabolism ontologies. Finally, 3 genes have not yet been classified: MARCKS, a myristoylated alanine-rich protein kinase C substrate; ENSA, an alpha endosulfine; and, TITF1, thyroid transcription factor 1.

**Figure 2:**
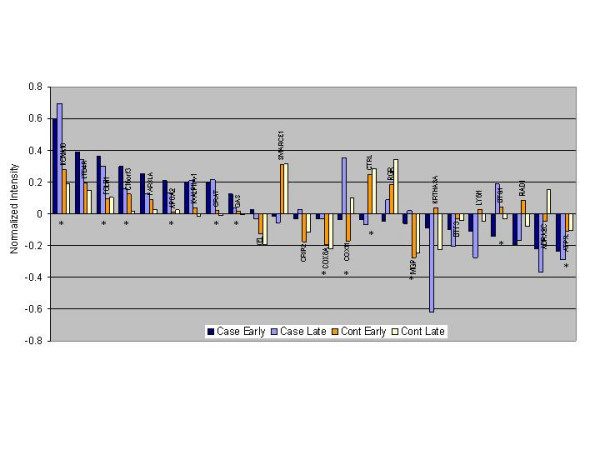
Comparison of genes that were expressed at similar levels during early and late time points for cases (blue bars) but were different for controls (orange bars). There were 24 of 636 genes that were differentially expressed. Half of these genes have known mitochondrial function and are marked with an asterisk.

To determine why people with post-infective fatigue responded differently to acute EBV infection, we next compared overall gene expression profiles including both early and late time points in cases and controls. Twenty-four genes were expressed at similar levels in cases at both the early and late time points but were significantly different from controls (Figure 2). Of these, 22 could be classified with the molecular function of binding, transporter, catalytic and signal transducer activity. More specifically, 12 of 24 could be associated with mitochondrial functions including fatty acid oxidation, apoptosis, DNA repair and mitochondrial membrane.

**Figure 3:**
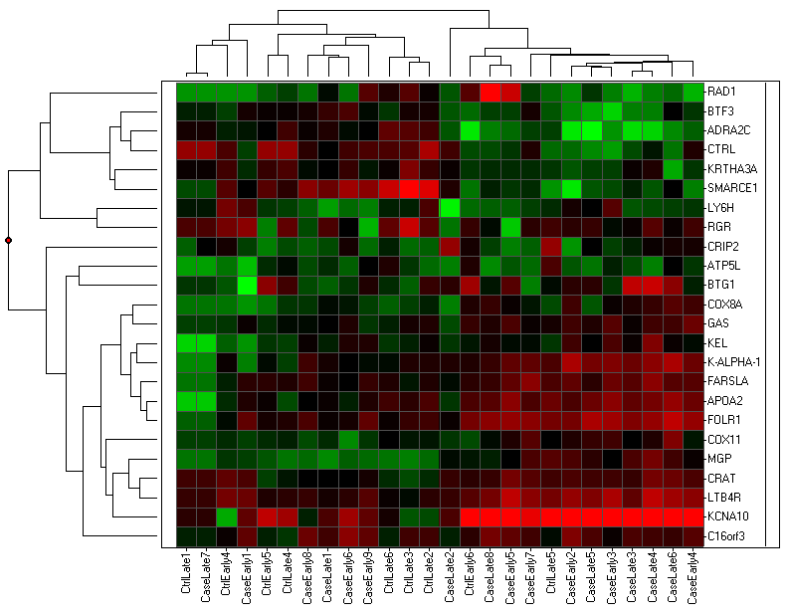
Cluster analysis of 24 differentially expressed genes of all samples from cases and controls at both early and late time points. The samples separated into 2 major groups; one containing most of the case samples and the other containing both cases and controls. The genes also grouped into 2 major groups; one that contained most of the mitochondrial function genes that were upregulated in cases and the other contained cell cycle checkpoints and DNA replication genes that were down regulated in the case group.

Cluster analysis of these 24 genes resulted in 2 major groups (Figure 3). One group was composed predominately of case samples (11/13; 85%) while the other group contained 6 case samples and 10 control samples. There were also 2 major gene groups, one with 17 genes and the other with the remaining 7 seven genes. In the larger gene group, 14 of 17 (82%) were up regulated in the case group and of these, 9 (9/14; 64%) were genes associated with mitochondrial functions. In the smaller group, these 7 genes were involved in cell cycle checkpoints and DNA replication and were down regulated in the case group.

## Discussion

EBV infection in teenage or adult life commonly results in the symptomatic illness known as infectious mononucleosis, which is marked by fever, pharyngitis and lymphadenopathy. Infectious mononucleosis typically has a febrile phase lasting 10 to 14 days. In most infections, a strong humoral and cellular response is mounted to control the virus. However, approximately 10% of subjects with EBV-associated infective mononucleosis remain sick for at least one year [24,25]. It has recently been shown that a small proportion of people remain sick with chronic fatigue syndrome up to 4 years following acute infectious mononucleosis [26]. This pilot study used gene expression profiling to determine whether failure to recover reflected altered gene expression associated with an altered host response to the acute EBV infection.

The subjects who did not recover from infectious mononucleosis had altered gene expression response during the early phase of EBV infection compared to those who subsequently recovered uneventfully. Several of the genes that were differentially expressed in cases were involved in immune response (ISG20; interferon stimulated gene), apoptosis (DNAJB2; DnaJ (Hsp40) homolog and CD99) and cell cycle (CDK8; cyclin-dependent kinase 8 and E2F2; E2F transcription factor 2) functions. It has recently been shown that EBV exploits these natural antiviral and cellular defense mechanisms to promote its survival and persistence in infected B cells [27]. In addition, efficient replication of the EBV genome is known to involve host cell cycle-associated proteins [28] including those encoded by three of the cell cycle genes identified here; CDK8 (cyclin-dependent kinase 8), E2F2 (E2F transcription factor 2) and ACTN2 (actinin, alpha 2). The transmembrane and epithelial adhesion protein, COL17A1 (collagen type XVII, alpha 1), that was differentially expressed, is shed from the cell surface by matrix metalloproteases (MMP) [29]. EBV proteins are known to regulate MMPs potentially altering the viral life cycle and cell motility [30]. KRTHB3 (basic keratin 3), MUC2 (mucin 2), and PCOLN3 (type III procollagen N-endopeptidase) all have altered expression in EBV-associated gastric carcinomas [31]. Since the majority of these genes are regulated during EBV replication, the fact that these were differentially expressed in cases potentially implicates a failure of the host to adequately control viral replication.

To determine why the response to EBV varied between cases and controls, both early and late time points in the natural history were compared. Of the 24 differentially expressed genes, half are involved in mitochondrial functions including fatty acid metabolism (CRAT; carnitine acetyltransferase, APOA2; apolipoprotein A-II), apoptosis (BTG1; B-cell translocation gene 1, FOLR1; folate receptor 1, CTRL; chymotrypsin-like), and mitochondrial membrane function (COX8A; cytochrome c oxidase subunit VIII, COX11; cytochrome c oxidase assembly protein, KCNA10; potassium voltage-gated channel, MGP; matrix Gla protein; ATP5L; ATP synthase). Several EBV-associated proteins, such as BRLF1, BHRF1, EBNA and LMP1 have been shown to interfere with fatty acid metabolism, mitochondrial function and apoptosis pathways [32-35].

Because mitochondria serve several prominent and essential cellular functions such as energy homeostasis, signaling and apoptosis, it is reasonable to surmise that alterations in mitochondrial function could affect immune function. In fact, viral and immune factors have been evaluated in these same subjects and it was found that post-infective fatigue was associated with an altered pattern of humoral immune response against EBV antigens [36]. An increase in the number of apoptotic peripheral blood cells has also been demonstrated in patients with chronic fatigue syndrome [37,38]. Although the numbers of subjects in this pilot study was small and only 3,800 genes were evaluated, our preliminary results implicate mitochondrial dysfunction as a plausible physiologic perturbation in post-infective fatigue.

## Conclusion

A small proportion of people that develop infectious mononucleosis following EBV infection remain sick with chronic fatigue syndrome. Gene expression profiling was used to determine whether failure to recover reflected altered gene expression. The subjects who did not recover from infectious mononucleosis had altered gene expression response during the early phase of EBV infection compared to those who subsequently recovered uneventfully. There were several differentially expressed genes identified including gene involved in the immune response, apoptosis and the cell cycle. A comparison of gene expression profiles early and late following EBV infection revealed that those who did not recover had differentially expressed genes implicating mitochondrial perturbations with fatty acid metabolism, mitochondrial function and apoptosis pathways.

## Competing interests

The author(s) declare that they have no competing interests.

## Authors' contributions

AL and IBH conceived and implemented this study and contributed to the drafting and finalization of this manuscript. SDV, TW and BC conducted the laboratory research, that analysis and the drafting and finalization of this manuscript. WCR advised on study design and analytical issues and assisted in the drafting and finalization of this manuscript.

## Pre-publication history

The pre-publication history for this paper can be accessed here:


